# Novel R Pipeline for Analyzing Biolog Phenotypic Microarray Data

**DOI:** 10.1371/journal.pone.0118392

**Published:** 2015-03-18

**Authors:** Minna Vehkala, Mikhail Shubin, Thomas R Connor, Nicholas R Thomson, Jukka Corander

**Affiliations:** 1 Department of Mathematics and Statistics, University of Helsinki, Helsinki, Finland; 2 Pathogen Genomics, Wellcome Trust Sanger Institute, Cambridge, United Kingdom; 3 Cardiff University School of Biosciences, Cardiff University, Cardiff, Wales, United Kingdom; 4 Department of Infectious and Tropical Diseases, London School of Hygiene and Tropical Medicine, London, United Kingdom; Cairo University, EGYPT

## Abstract

Data produced by Biolog Phenotype MicroArrays are longitudinal measurements of cells’ respiration on distinct substrates. We introduce a three-step pipeline to analyze phenotypic microarray data with novel procedures for grouping, normalization and effect identification. Grouping and normalization are standard problems in the analysis of phenotype microarrays defined as categorizing bacterial responses into active and non-active, and removing systematic errors from the experimental data, respectively. We expand existing solutions by introducing an important assumption that active and non-active bacteria manifest completely different metabolism and thus should be treated separately. Effect identification, in turn, provides new insights into detecting differing respiration patterns between experimental conditions, *e.g.* between different combinations of strains and temperatures, as not only the main effects but also their interactions can be evaluated. In the effect identification, the multilevel data are effectively processed by a hierarchical model in the Bayesian framework. The pipeline is tested on a data set of 12 phenotypic plates with bacterium *Yersinia enterocolitica*. Our pipeline is implemented in R language on the top of opm R package and is freely available for research purposes.

## Introduction

Recent advances in measurement technology have produced an explosion of information on the genetic and phenotypic characteristics of bacteria. Inexpensive whole-genome sequencing has just begun to shape our detailed understanding of intercontinental transmission patterns and how rapidly bacterial populations can respond to environmental pressures resulting from vaccines and antibiotics.

Biolog Phenotype MicroArrays (PMs) are commercially available microplates utilized in genomic research [1–3]. Specifically, they are widely used in the characterization of metabolic activity. As a simple example, metabolic alterations between diseased and healthy cell lines can be revealed using this technology [4]. One way to utilize PMs is to establish a link between a genotype and phenotype. For example, the function of a gene correlated with a disease state or another factor of interest, can be determined by inoculating a sample of interest into PMs [5, 6].

The tests on microplates represent a comprehensive array of over 2000 cellular phenotypes including the ability to catabolize basic elemental nutrients, such as carbon, nitrogen, phosphorus, and sulfur based substrates, as well as drug and chemical sensitivities. Metabolic capabilities of cells are identified via redox reactions associated with cellular respiration. Respiration of the substrate in each well leads to the reduction of a tetrazolium dye and a concomitant measurable colour change. If a bacterium lacks the genes to catabolize that substrate or if respiration is slowed or stopped, no or less dye is reduced and therefore no colour is observed. It is possible to measure the reduction of the dye over several hours of incubation and to plot a profile representing the kinetics of that substrate utilization over time. We refer to these kinetic plots as metabolic profiles for the purposes of this study since we used substrate utilization to validate our approach.

Although the Biolog PM system has been available for over a decade, the development of statistical analysis methodology has not been as intensive as for other, more common genomic microarray platforms such as Affymetrix and Illumina. Therefore, the majority of Biolog data analyses are still performed using Biolog PM in-house developed procedures, standard explorative techniques such as PCA [7], or basic statistical tests involving the comparison of two groups (ANOVA). Multivariate non-model-based statistical techniques, such as PCA and clustering, are certainly useful in detecting groups of substrates with similar metabolic profiles and identifying substrates whose profiles differ between treatments [7]. However, the existing analysis methods can be further improved. Recently, Vaas *et al*. (2012) introduced a comprehensive R package, opm, for analysis of PM data.

Typically, Biolog phenotype microarrays are produced in technical replicates in order to increase the power in detecting differences between metabolisms under varying conditions. However, replicates of the same experimental conditions may be incomparable, *e.g*. due to differing laboratory conditions, or differences in the array or sample quality. These kinds of artefacts typically affect all the measurements on a plate by consistently decreasing or increasing the metabolic signals. Since the outcome of interest can be blurred by these array effects, they need to be identified and removed before constructing further statistical analyses. However, previous research has been limited in terms of accounting for the array effect. In general, when genomic microarrays are considered, normalization (*i.e*. adjusting the measurements to make the samples comparable) is a standard procedure. However, disagreement exists concerning the necessity of normalizing PMs. Some researchers divide the original or background subtracted metabolic signals by the average well colour development (AWCD) [8–10], while others are cautious about this methodology [7, 11]. We, in turn, separate wells producing positive metabolic signals from wells showing no signal, while normalizing.

In this article, our aim is to introduce efficient and meaningful statistical methods for analysis of PM data that go beyond the methods suggested in earlier works. Specifically, we focus on normalization, grouping of PM data and effect identification. To systematicize analysis of PM data similar to oligonucleotide expression arrays, we have sought to develop a model-based alternative to the widely used non-model-based methods, which most often rely on several *ad hoc* criteria for drawing conclusions. For example, we used curve fitting, the potential of which in characterizing PMs has been recognized decades ago [7, 12, 13] but not implemented as a comprehensive framework until recently [14]. According to our findings, a logistic model appears appropriate for characterizing the metabolic profiles of PM data and in this article we demonstrate, how the logistic model can be utilized during the multi step analysis of PM data to reach the above-mentioned desiderata.

## Materials and Methods

We use a recently published phenotype microarray data set [15] to illustrate the functionality of our analysis pipeline. Seven bacterial strains of the bacterium *Yersinia enterocolitica* were cultured on PM1, 2A, 3B and 4A and incubated for 48h at 28°C in the OmniLog Incubator/Reader. We limit our analysis to two strains, 53/03 and 8081c, on plate PM1. Additionally, Reuter *et al*. provide us data for the corresponding strains incubated at 37°C, the preparation of which is similar to strains incubated at 28°C.

Our methods are implemented by the statistical software R and can be used on top of the opm R package [14]. R scripts for running the example pipeline are available through http://www.helsinki.fi/bsg/software/R-Biolog. Data can also be accessed with doi:10.5061/dryad.r98g7.

The analysis pipeline described below is divided into three steps: (1) grouping, (2) normalization and (3) effect identification. A graph illustrating the pipeline is shown in S1 Fig.

### Grouping

**Fig 1:**
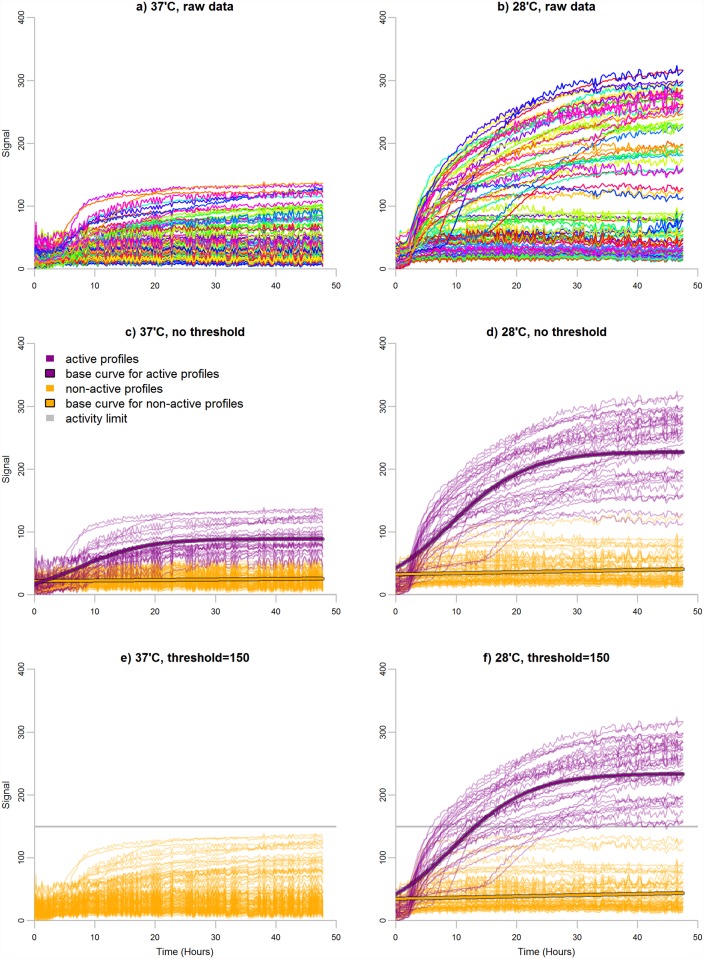
Grouping of PM profiles. Metabolic profiles of *Yersinia enterocolitica*, strain 8081c, plate PM01, at two different temperatures: 37°C (panels a, c, e) and 28°C (panels b, d, f). Panels (a) and (b) present the raw data. Each line represents the metabolic signals observed in a single well. Time in hours and the strength of the metabolic signals are represented on the x- and y-axes, respectively. Grouping separates active profiles from the non-active ones by using the EM algorithm. Panels (c) and (d) present the grouped data when no threshold is applied. Active profiles are shown in purple and non-active in orange. Base curves illustrating the average patterns of the two groups are highlighted. The EM algorithm is unable to handle the array with all non-active profiles (panel c), as it always identifies two clusters. Panels (e) and (f) present the data grouped by applying a threshold of 150 (shown as the grey horizontal line). The array with only non-active profiles is successfully identified (panel e), while the grouping on the other array stays almost unaffected (panel f).

One of the most central questions related to the use of PMs is measuring whether bacteria are able to utilize a specific substrate under given experimental conditions. The aim of our grouping step is thus to identify two groups of wells, one of which shows positive metabolic signals and the other no signals. Wells associated with a positive signal serve as a sufficient energy supply enabling cell respiration. On the contrary, wells with no signal lack the ability to catabolize that substrate. We refer the profiles in the positive and no-signal groups as active and non-active, respectively. Similarly, if bacteria produce positive metabolic signals or no signals, we say the bacteria are either active or non-active, respectively. Fig. 1 represents an example of grouped metabolic profiles.

To perform the grouping we use an important assumption that active and non-active bacteria manifest completely different metabolism and thus should be described with different models. The metabolic profiles produced by active bacteria are modelled with a logistic growth model (highlighted purple lines in Fig. 1c, d and f)

$$\begin{array}{c} F_{t}^{active}=\frac{Asym}{1+\exp(\frac{xmid-t}{scale})}, \end{array}$$ (1)

where *t* ∈ *T* is a time point, *T* is the set of all time points at which the metabolic signal is observed (*e.g*. for the data analysed in this research *T* = {0, 0.25, 0.5, …, 47.75} hours), *Asym* is the signal as *t* → ∞, *xmid* is the time at which the signal is *Asym*/2, and *scale* is the inverse of the maximum growth rate. The metabolic profiles produced by inactive bacteria are modeled with a line (highlighted yellow lines in Fig. 1c, d and f)

$$\begin{array}{c} F_{t}^{non-active}=b_{0}+b_{1}*t, \end{array}$$ (2)

where *b*_0_ is a starting level and *b*_1_ is a slope.

The grouping is performed separately for each array. The user may specify a threshold value (see Fig. 1e and f). If a metabolic profile is below the threshold (*i.e*. the metabolic signal is lower than the threshold at each time point) it is peremptorily labeled non-active. If all the profiles are labeled this way, the downstream grouping algorithm is not utilized (see Fig. 1e). Otherwise, or if a threshold is not specified, profiles are iteratively clustered with the Expectation-Maximization (EM) algorithm [16, 17]. The user-given threshold is utilized to specify an initial grouping, so that profiles below the threshold are assigned into the non-active group, while the rest are labeled active. If the threshold is not specified, the initial grouping is randomly chosen. After initialization the algorithm proceeds iteratively until convergence or maximum number of iterations is reached. At each iteration, two steps: expectation and maximization, are performed. In the maximization step, a mixture of the logistic curve (1) and line (2) is fitted to profiles:

$$\begin{array}{c} Signal_{t}=\left(1-Group\right)*F_{t}^{non-active}+Group*F_{t}^{active}+\epsilon_{t}, \end{array}$$ (3)

where *Signal*_*t*_ is the metabolic signal observed at time point *t*, *Group* is a binary (0, 1) grouping covariate and *ϵ* is Gaussian noise. The fitting of (3) is done by a modification of the Levenberg-Marquardt algorithm provided by the minpack.lm package of the R software (www.r-project.org, version 3.0.3). If the algorithm fails to fit the logistic model to the active profiles, then linear model is fitted. If the procedure results in fitted values below zero, these are truncated to zero since negative values for the metabolic signal do not biologically make sense. In the expectation step, each profile is assigned to the most probable group. Profiles below the user-given threshold are always labeled non-active.

### Normalization

**Fig 2:**
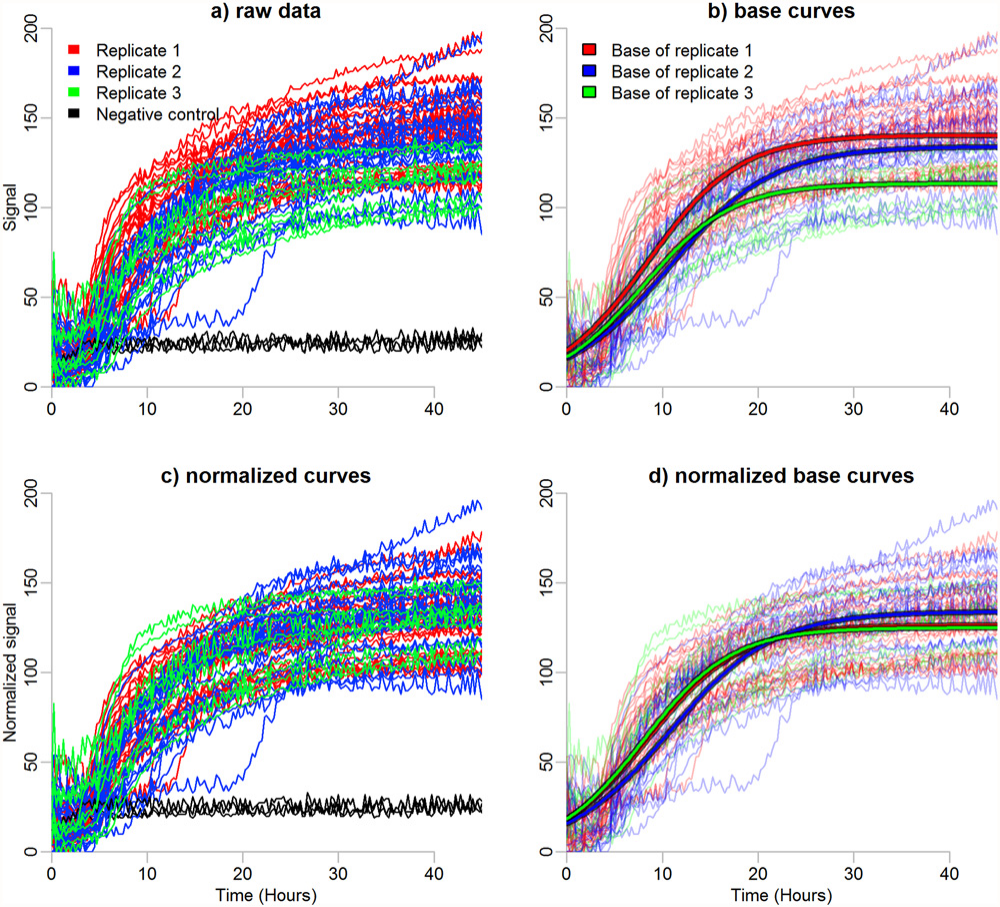
Normalization of PM profiles. Metabolic profiles of three replicates of *Yersinia enterocolitica*, strain 8081c, plate PM01, at temperature 37°C. Only the profiles classified active are shown. (a) Raw data. Time in hours and the observed metabolic signals are represented on the x- and y-axes, respectively. The first replicate (red lines) shows persistently stronger signals than the second replicate (blue lines). The third replicate (green lines) shows weaker signals than the first two. The negative controls do not indicate the metabolic signals to differ between the replicated arrays (black lines). (b) Base curves fitted into the raw data. Base curves emphasize the differences between the replicates. (c) Normalized data. The second replicate is used as a reference. Normalization is performed based on the non-stabilized grouping. (d) Base curves of the normalized data.

The purpose of normalization is to make arrays comparable with each other. In the case of PMs, the arrays are not comparable as such since regardless of identical experimental conditions one array can produce systematically stronger or weaker signals than the others, similar to oligonucleotide arrays. These global differences among arrays may be, *e.g*. due to array quality or errors in sample preparation. Normalization is needed to detect and remove such systematic variation derived from other sources than differential responses to the experimental conditions. A paradigm of an array effect and a proposed solution is illustrated in Fig. 2.

In the existing methods, all metabolic profiles, irrespective of the strength of the observed signal, are normalized either by dividing the raw metabolic signals by the overall average profile or by subtracting the background signal from the raw values [9, 10, 18]. These methods can cause biased estimates. For example, if there are few active profiles on an array, the average profile is dominated by the behaviour of the non-active profiles.

We suggest normalizing the active and non-active metabolic profiles separately. For each plate we compute a base active curve and compare it to the base active curves from the experimental replicates, *i.e*. plates with the same bacterial strain observed under the same conditions (see Fig. 2a and b). Then the procedure is repeated for the non-active profiles.

The information contained in replicated measurements is also used for stabilizing the profiles. Stabilization is required if profiles of corresponding wells on replicated arrays are labeled inconsistently. Usually the replicated measurements resemble each other to a considerable degree and their group labels are identical. However, if there is either substantial technical noise or variation in the metabolism between replicated measurements, replicates could have different activity labels. If one profile is labeled differently from the others, it is likely to represent a false signal since the same effect is not observed in the other replicates. To stabilize, the profiles are given the label they have in a user-defined proportion of the replicates. Stabilization removes false signals and eases downstream analysis, as after stabilization replicates of the same condition have the same number of active and non-active profiles.

The base curves for the active and non-active groups are defined by fitting a logistic curve (1) to the active, and a line (2) to the non-active metabolic profiles of an array. Similar to the grouping procedure, the base curves are fitted by a modification of the Levenberg-Marquardt algorithm provided by the minpack.lm package of the R software. If the algorithm fails to fit the logistic model to the active profiles, then linear model is fitted.

After fitting the base curves, the normalization is accomplished by defining one of the arrays as a reference, leaving it unchanged and adjusting the metabolic profiles of the other arrays to the level of the reference. We select the reference array to be the replicate closest to other replicates (replicate 2 in Fig. 2b). Here, the distance of an array to other arrays is defined as the sum of the Euclidean distances between the base curves of the arrays. Reference arrays for the active and non-active groups are selected separately.

For a non-reference array the signal is multiplied by a normalization factor (NF), which is the ratio of the base curve of the reference and the base curve of the array in question:

$$\begin{array}{c} Normalized_{t,j,k}=Signal_{t,j,k}*NF_{j,k}=Signal_{t,j,k}\frac{\sum_{\tau \in T}Base\_curve_{\tau ,R(k),k}}{\sum_{\tau \in T}Base\_curve_{\tau ,j,k}}, \end{array}$$ (4)

where *t* ∈ *T* is time point, *T* is the set of time points at which the metabolic signal was observed, *j* is the index of the normalized array, *R*(*k*) is the index of the reference array and *k* ∈ {*active*, *non*—*active*}. If normalization results in negative values for the signal, these are truncated to zero. After the normalization, replicated experiments are more compatible and experiments performed under different conditions more reliably comparable (see Fig. 2c and d).

### Effect identification

**Fig 3:**
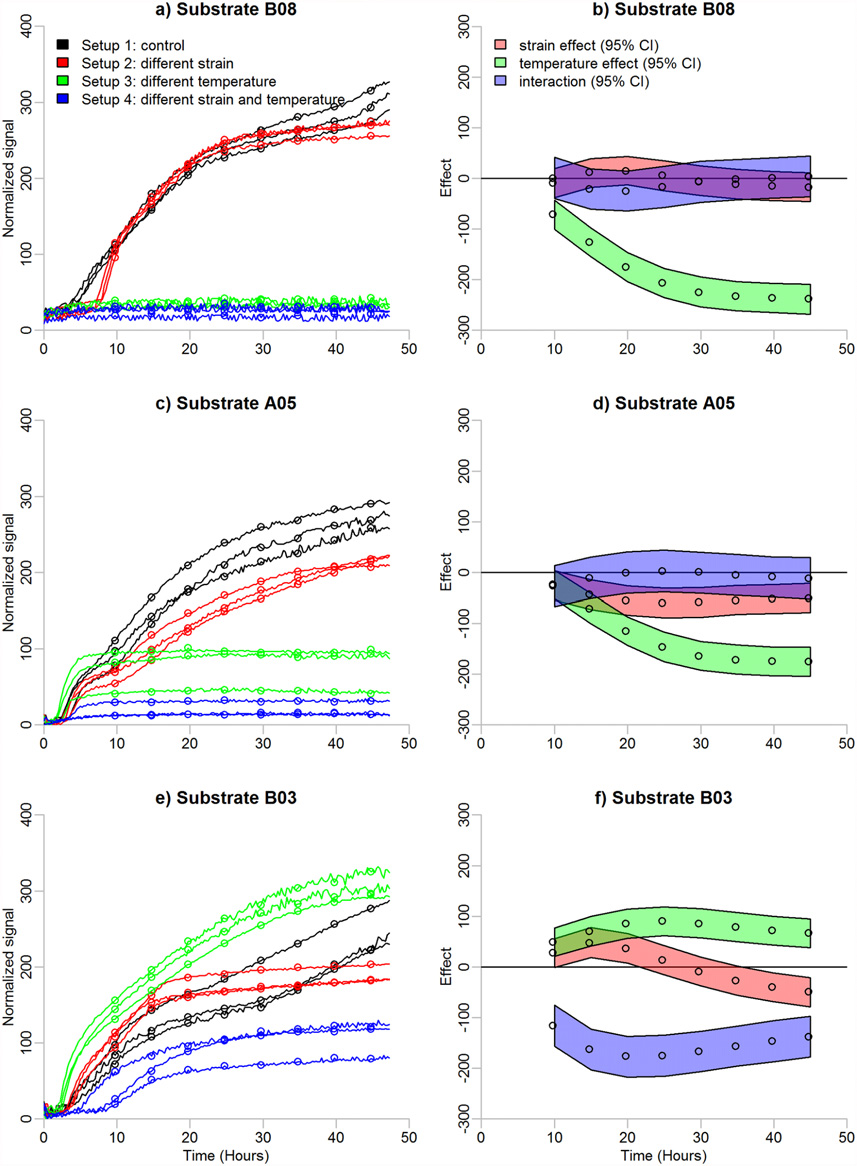
Effect identification of PM profiles. Panel (a) shows metabolisms of the bacterium *Yersinia enterocolitica* in substrate B08, panel (c) in substrate A05 and panel (e) in substrate B03. Experiments are performed under four different experimental conditions which are represented by different colours. Measurements under each experimental condition are replicated three times. Time in hours and the normalized metabolic signals are represented on the x- and y-axes, respectively. A line or logistic curve is fitted to each profile according to its activity status. The dots illustrate thinned sequences of the fitted values. Three effects are of interest: main effects for strain and temperature, and their interaction. Effects are estimated as the mean values of Markov chains produced by a variance analysis model. Panels (b), (d) and (f) show the estimates of the effects and their 95% Bayesian credibility intervals.

In the third step of the analysis pipeline, we provide a method for determining whether metabolism changes significantly when experimental conditions (*e.g*. temperature, strain or genetic background) are modified. In contrast to the array level analyses represented in the previous steps, this analysis step addresses profiles on substrate level. We do not only test the main effects of conditions but also their interaction which has not been considered in earlier PM analysis techniques [9, 14, 19]. Including the interaction term in the analysis allows the inspection of the level of one factor affecting the metabolic signals produced by the other factor (see Fig. 3).

To apply the effect identification algorithm, the user should define one experimental condition as a control (black lines in Fig. 3a, c and e). All the other experimental conditions are compared against the control treatment. We assume that the experimental conditions are defined by two factors: A and B. Three effects are of interest: 1) the main effect of factor A, 2) the main effect of factor B, and 3) the interaction of factors A and B.

We start by removing noise from the data by fitting the logistic (1) and linear (2) models to the active and non-active profiles, respectively. Similar to the grouping and normalization procedures, the fitting of (1) and (2) is done by a modification of the Levenberg-Marquardt algorithm provided by the minpack.lm package of the R software. If the algorithm fails to fit the logistic model to an active profile, then linear model is fitted.

In the earlier steps, the fitted profiles represented the average behaviour for a group of profiles: either active or non-active profiles in the grouping step (highlighted lines in Fig. 1c, d and f), and different replicates in the normalization step (bases in Fig. 2b and d). Now, the models describe individual profiles (dots in Fig. 3a, c and e).

We consider two aspects: aggregated effects of the conditions on the metabolic profiles over time (*i.e*. averaged estimates), as well as the changes in dynamics between individual time points (*i.e*. time point-wise estimates). To reduce the computational complexity, we study a subset of time points T* ⊂ *T* which can be chosen by the user or set to a default option. Fitted values at the chosen time points serve as data for a Bayesian hierarchical variance analysis.

For a fixed substrate, the data consist of *N* profiles, representing the behaviour of the bacteria in the same well but on different arrays. These data are given in the form of *y*_*i*, *t*_, *a*_*i*_ and *b*_*i*_. Here, *i* ∈ {1, …, *N*} is the index of the array, *t* ∈ T* ⊂ *T* is a time point from the given subset of time points, *y*_*i*, *t*_ is the value of the model (1 or 2) fitted into profile *i*, and *a*_*i*_ ∈ {1, …, *Levels*_*A*_} and *b*_*i*_ ∈ {1, …, *Levels*_*B*_} are the levels of factors A and B for the *i*th array, *Levels*_*A*_ and *Levels*_*B*_ are the number of levels for these factors. Without loss of generality, the control treatment is given values *a* = 1 and *b* = 1.

For the Bayesian hierarchical model the likelihood is defined in the following way:

$$\begin{array}{c} y_{i,t}∼Normal\left(\mu_{i,t},\sigma^{2}\right) \end{array}$$

$$\begin{array}{c} \mu_{i,t}=\left\{\begin{array}{ccc} \zeta_{t} & \;\text{if}\; & a_{i}=b_{i}=1\\ \zeta_{t}+\alpha_{a_{i},t} & \;\text{if}\; & a_{i}\neq 1,b_{i}=1\\ \zeta_{t}+\beta_{b_{i},t} & \;\text{if}\; & a_{i}=1,b_{i}\neq 1\\ \zeta_{t}+\alpha_{a_{i},t}+\beta_{b_{i},t}+\gamma_{a_{i},b_{i},t} & \;\text{if}\; & a_{i},b_{i}\neq 1 \end{array}\right. \end{array}$$

In the above model formulation, the response variable *y*_*i*, *t*_ is a continuous random variable following the normal distribution with mean *μ*_*i*, *t*_ and variance *σ* ^2^. The composition of the mean *μ*_*i*, *t*_ is dependent on the experimental conditions. The parameter *ζ*_*t*_ is an effect specific to the control treatment at time *t*; *α*_*a*_*i*_, *t*_ and *β*_*b*_*i*_, *t*_ are effects specific to values of *a*_*i*_ ≠ 1 or *b*_*i*_ ≠ 1, and *γ*_*a*_*i*_, *b*_*i*_, *t*_ is the interaction effect.

Uninformative priors are implied for *ζ*, *α*, *β* and *γ*:

$$\begin{array}{cc} \zeta_{t} & ∼Uniform(0,400)\\ \alpha_{a\neq 1,t} & ∼Normal(0,1000)\\ \beta_{b\neq 1,t} & ∼Normal(0,1000)\\ \gamma_{a\neq 1,b\neq 1,t} & ∼Normal(0,1000) \end{array}$$

This comparison is applied to every substrate. The only parameter that is shared between the substrates is the model error *σ* ^2^. The user can control the sensitivity of the analysis by defining the magnitude of *σ* ^2^. By choosing a small model error, small differences between the experimental setups can be identified. On the contrary, a larger model error allows a more liberal analysis.

The posterior distribution of each effect parameter is simulated by WinBUGS (http://www.mrc-bsu.cam.ac.uk/software/bugs/the-bugs-project-winbugs, version 1.4.3) run via R software. The average values of the simulated chains can be taken as the estimates for the effects of interest (see Fig. 3b, d and f).

## Results

The data set and the r scripts used for the following analyses are available through http://www.helsinki.fi/bsg/software/R-Biolog. Data can also be accessed with doi:10.5061/dryad.r98g7. Table 1 presents a summary of the composition of the data. A full description of the data is given in the supplements (S2 and S5 Figs). Analysis proceeds in the order of the three main steps presented in the previous section.

**Table 1:** Data composition and results of grouping and normalization.

Data				Active profiles		Normalization factor	
Array id	Setup id	Temperature	Strain	Non-stabilized	Stabilized	Active	Non-active
1	1	28	53/03	43	43	1.06	1.06
2				42		1	1
3				45		0.96	0.96
4	2	28	8081c	42	42	1.09	1.02
5				40		1	1
6				40		0.97	0.90
7	3	37	53/03	30	33	0.97	1
8				33		1	0.87
9				37		1.08	1.24
10	4	37	8081c	28 (30)	25	0.90	1
11				25 (29)		1	1.01
12				10 (25)		1.10	0.87

The data set consists of 12 arrays divided into 4 setups according to the possible combinations of temperature and strain. The 96 metabolic profiles of each array are grouped into active and non-active categories by applying a threshold of 100. The number of active profiles on each array is shown. Numbers in brackets for the Setup 4 show the alternative results when no threshold is given. Stabilized grouping is steady over replicates. In the normalization, non-stabilized grouping is utilized. Normalization factor is the ratio of the base curves of the reference and the array in question. Reference arrays have a normalization factor of one.

### Grouping

The metabolic profiles shown in S2 Fig are grouped into active and non-active with the threshold for activity set to 100. The results of grouping are specified in Table 1 and in S3 and S6 Figs. At temperature 28°C (Setups 1 and 2) on average 44% of the 96 profiles are active. At 37°C, approximately 33% of the profiles are active for strain 53/03 (Setup 3), and only 22% for strain 8081c (Setup 4).

We tested two different thresholds to examine the impact of the threshold on the grouping. However, defining the threshold to 0 or to 100 has a minimal effect on the results. In total, only 21 profiles change labels from active to non-active when the threshold is changed from 0 to 100. All these profiles belong to the experimental Setup 4 (the numbers in brackets in Table 1). The metabolic signals on these arrays are weak, even for the active profiles (see S2 Fig: arrays 10, 11 and 12). Thus, applying the EM algorithm without a threshold for these arrays results in misclassifications, as clearly non-active profiles are labeled active (compare Fig. 1c with arrays 10, 11 and 12 in S3 Fig).

### Normalization

The results of normalization are shown in Table 1 and in S4 and S7 Figs. Arrays are multiplied by normalization factors from the interval of [0.87, 1.24]. The normalization of the active profiles on the arrays 10, 11 and 12 (Setup 4) is detailed in Fig. 2. Considering Setup 4, the largest normalization factor (1.10) is applied to the active profiles of the array 12. This is reasonable, as array 12 has significantly weaker metabolic signals than its replicates (compare green line to red and blue lines in Fig. 2b).

### Effect identification

The results of effect identification for the complete data set are shown in S8 and S9 Figs. The results for substrates B08, A05 and B03 are detailed in Table 2 and in Fig. 3.

**Table 2:** Effect identification: averaged estimates.

Substrate	Effect		Mean	CI
B08	Control:	28°C, 53/03	225.10	(217.90, 232.50)
	Strain:	28°C, 8081c	−3.32	(−13.46, 6.57)
	Temperature:	37°C, 53/03	−188.80	(−199.20, −178.60)
	Interaction:	37°C, 8081c	−8.42	(−22.54, 5.72)
A05	Control:	28°C, 53/03	206.10	(198.80, 213.70)
	Strain:	28°C, 8081c	−49.77	(−60.06, −39.28)
	Temperature:	37°C, 53/03	−130.10	(−140.50, −119.85)
	Interaction:	37°C, 8081c	−7.31	(−22.24, 7.02)
B03	Control:	28°C, 53/03	167.20	(160.10, 174.30)
	Strain:	28°C, 8081c	−0.10	(−9.81, 9.85)
	Temperature:	37°C, 53/03	74.84	(65.00, 84.86)
	Interaction:	37°C, 8081c	−154.50	(−168.90, −141.00)

Mean is the averaged estimate over time points. CI is the 95% credibility interval.

Substrate B08 represents an example of a substrate related to a significant effect for temperature. The effect has a decreasing trend (green section in Fig. 3b), meaning that the difference between the metabolic signals measured at 28°C and 37°C increases over time (*i.e*. in Fig. 3a, the difference between the black and green lines increases along time). On average, metabolic signals detected at 37°C are 189 units weaker than signals at 28°C (see Table 2). Since the interaction effect does not differ from zero at any of the time points (blue section in Fig. 3b) and thus is not significant, the difference between signals at different temperatures does not depend on the strain (*i.e*. in Fig. 3a, the difference between the black and the green lines is the same as the difference between the red and the blue lines). The strain effect is not significant either (red section in Fig. 3b), meaning that there is no difference in the utilization of substrate B08 between the strains 53/03 and 8081c (*i.e*. in Fig. 3a, there is no difference between either the black and the red lines or between the green and the blue lines).

In comparison to substrate B08, substrate A05 is related to a significant strain effect, in addition to a significant temperature effect (*i.e*. in Fig. 3c, in addition to the black lines differing from the green ones, and the red differing from the blue ones, the red lines differ from the black, and also the blue from the green). Similar to substrate B08, the temperature effect decreases along time (green section in Fig. 3d), being on average 130 units lower at temperature 37°C than at temperature 28°C (see Table 2). The effect for strain is more negligible being on average −50.

For substrate B03, all the three effects are significant at most of the time points. Temperature effect is positive (green section in Fig. 3f) while interaction effect is negative (blue section in Fig. 3f). Strain effect is positive at the beginning of the process and negative at the end (red section in Fig. 3f) due to that at 28°C the reaction of strain 8081c (red lines in Fig. 3e) is faster than the reaction of strain 53/03 (black lines in Fig. 3e) at the early time points, but the signals of strain 8081c settle earlier than the signals of strain 53/03. In contrast to the conclusions made based on the time point-wise estimates, averaging over time points produces an insignificant estimate for the strain effect (see Table 2).

## Discussion

We present a novel statistical pipeline which allows for a comprehensive analysis of multi-factored PM experiments. The pipeline has three stages: 1) separating active Biolog PM metabolic profiles from the non-active ones, 2) making arrays comparable with each other by normalization and 3) comparing metabolisms under different experimental conditions using a Bayesian analysis of variance. To demonstrate the utility of our tools for analyzing PM data we use a data set containing 12 arrays measured under four experimental conditions. A small-scale data set is chosen for purpose of easy illustration, however no hinders exist for analyzing larger data sets with a wide range of experimental settings.

In the grouping step, we use the EM algorithm to separate active metabolic profiles from the non-active ones. It is possible to have a plate with only non-active bacteria (for example, see Fig. 1, panel e). Out of the box, the EM algorithm cannot handle these arrays correctly, as it will always identify two groups: active and non-active. The proposed pipeline solves this problem by allowing the user to define an activity threshold. Defining a meaningful threshold is particularly important when the metabolic signals on an array are weak. In such a case, the threshold allows all the profiles to be labeled non-active. The EM algorithm can also be replaced by simpler methods, such as 2-means clustering without significantly affecting the results.

The existing normalization methods for PM data, such as AWCD-based methods, process one plate at a time while we normalize the arrays over the experimental replicates. This also differs from the usual practise applied with gene expression data, where arrays are normalized over all samples [20]. If applied with a PM setting, this excessive normalization could blur informative variation introduced by different experimental conditions, where as comparison over replicates does not hide interesting information but is able to reveal array effects. In addition to exploiting the experimental replicates, we normalize profiles conditional on statistical grouping into wells showing either positive metabolic signal or no signal at all. Our method results in a statistically more sound and effective procedure since direct normalization of raw measurements with plate averages may be heavily affected by varying ratios of non-active metabolic profiles.

By default a stabilized grouping is used in the normalization, meaning that the same substrates are associated with active/non-active labels on replicated arrays. Comparison of the same substrates over replicates reflects array effects better than comparison of different substrates on replicated arrays. Often there is no significant difference between the normalization performed with or without the stabilization. Thus, the non-stabilized grouping can be used as well. However, if one replicate contains only non-active profiles, the stabilization is crucial since otherwise the evident array effect would remain undetected.

In the normalization procedure, one replicate acts as a reference. All the other replicates are compared against this reference array. In each experimental condition, the reference array is defined separately for the active and non-active profiles since the possible artefact on a plate might affect in a different manner the signals in these two groups. For example, in the group of active profiles one array might produce systematically weaker signals than the other arrays while no clear effect is visible among the non-active profiles.

A PM plate usually contains a negative control, *i.e*. growth media without bacteria. In the literature, there exists a mixed opinion whether to subtract the signals of the negative control from the other measurements on the same plate. The presented pipeline can be used in both cases. We applied our normalization procedure with both the original and background subtracted data (results for the background subtracted data are not shown). Essentially the comparison revealed, that the normalization is needed in both cases. Applying the background correction without normalizing does not suffice to make the arrays comparable since the negative control profile might not reveal the existing array effects (see Fig. 2a, black lines).

For the effect identification, it is possible to consider both time point-wise estimates and averages over the time points. Use of averages enables a fast inspection of the effects, whereas the time point-wise estimates allow a more detailed analysis. The two approaches complement each other, when used in tandem. Namely, effects that are positive at some time points and negative at others remain undetected by averaging since such effects cancel each other out. However, alternating effects can be revealed by point-wise estimates. See substrate B03 in Fig. 3 and Table 2 for an example. It should be noted that the numerical values for the estimates of the effects are dependent on the choice of the control treatment.

All the three main steps of our pipeline utilize fitting of two models, linear and logistic. The logistic model describes most of the active metabolic profiles with a high fidelity, whereas the simpler linear model efficiently describes the non-active, as well as the outlying shapes of the active profiles. In some PM data, it is possible to observe more complicated metabolic profiles, such as Michaelis-Menten-looking dynamics, period of linear growth, or a combination of these. A possible future ramification of the current method would thus be to model the time-series as mixtures of basic metabolic profiles using, for instance, time-lagged observations. To further improve the resolution of the method, more than two models could also be fitted to capture eventual different shapes of the active metabolic profiles.

Our pipeline has been implemented with R language and can be used as an add on to the opm R package which provides all the basic functionalities and statistical procedures for reading in, manipulating and visualizing Omnilog PM data. Additionally, minpack.lm R package is used for fitting logistic and linear curves. Effect identification, in turn, relies on WinBUGS program which is called from R to perform the Bayesian analysis of variance. The software code is available through www.helsinki.fi/bsg/software/R-Biolog.

## Supporting Information

S1 Fig Graph of the analysis pipeline. (PDF)

S2 Fig Raw PM profiles array-wise. (PDF)

S3 Fig Grouped PM profiles array-wise. (PDF)

S4 Fig Normalized PM profiles array-wise. (PDF)

S5 Fig Raw PM profiles substrate-wise. (PDF)

S6 Fig Grouped PM profiles substrate-wise. (PDF)

S7 Fig Normalized PM profiles substrate-wise. (PDF)

S8 Fig Effect identification. (PDF)

S9 Fig Effect identification. (PDF)
