# Supplementary material for: Novel R Pipeline for Analyzing Biolog Phenotypic Microarray Data
Source: PLoS One. 2015 Mar 18;10(3):e0118392. doi: 10.1371/journal.pone.0118392 (PMC4365023; doi:10.1371/journal.pone.0118392)
Supplement: S1 Fig — (PDF) [file pone.0118392.s001.pdf]

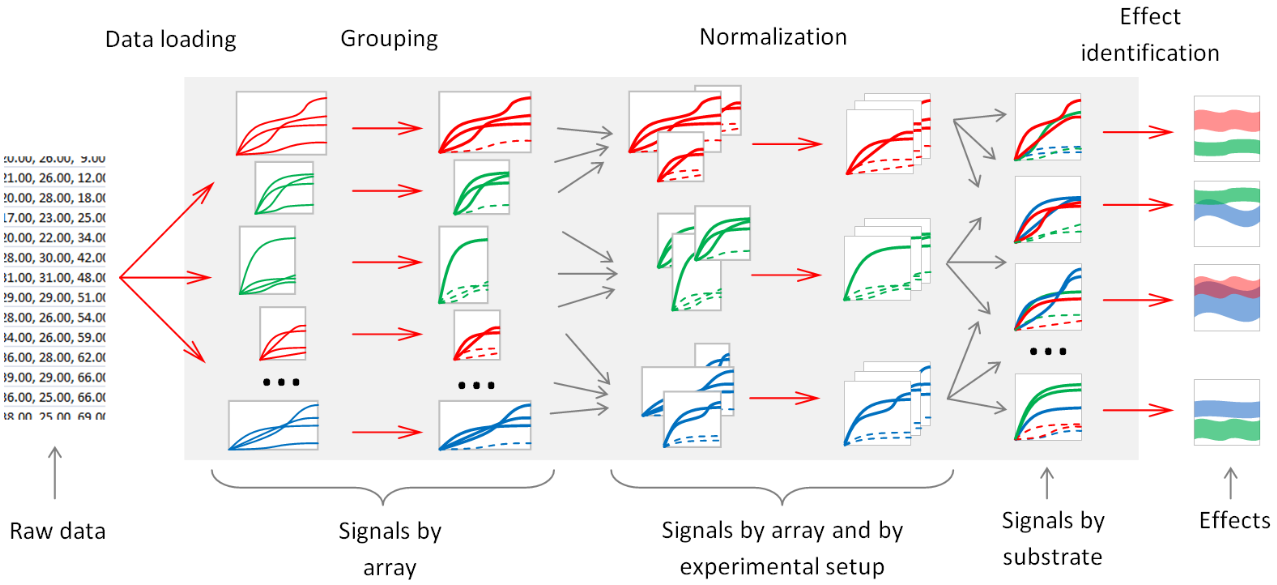

Figure S1. Graph of the analysis pipeline. The pipeline consists of three main steps: grouping, normalization and effect identification. Before these steps the raw numeric data is loaded into R workspace by using an R package called opm. Data plotted array-wise expose metabolic profiles over time. Different colours represent replicated measurements of an experimental setup. In the grouping step, the raw profiles are categorized by an EM algorithm into two groups which in the panels are illustrated by continuous and dashed lines. Next, the data is normalized. Normalization is performed for each experimental setup separately. Normalization makes the replicates of the same setup comparable with each other. Finally, effect identification reveals metabolic profiles differing between experimental conditions. This step is performed substrate-wise.
