# Supplementary material for: Novel R Pipeline for Analyzing Biolog Phenotypic Microarray Data
Source: PLoS One. 2015 Mar 18;10(3):e0118392. doi: 10.1371/journal.pone.0118392 (PMC4365023; doi:10.1371/journal.pone.0118392)
Supplement: S3 Fig — (PDF) [file pone.0118392.s003.pdf]

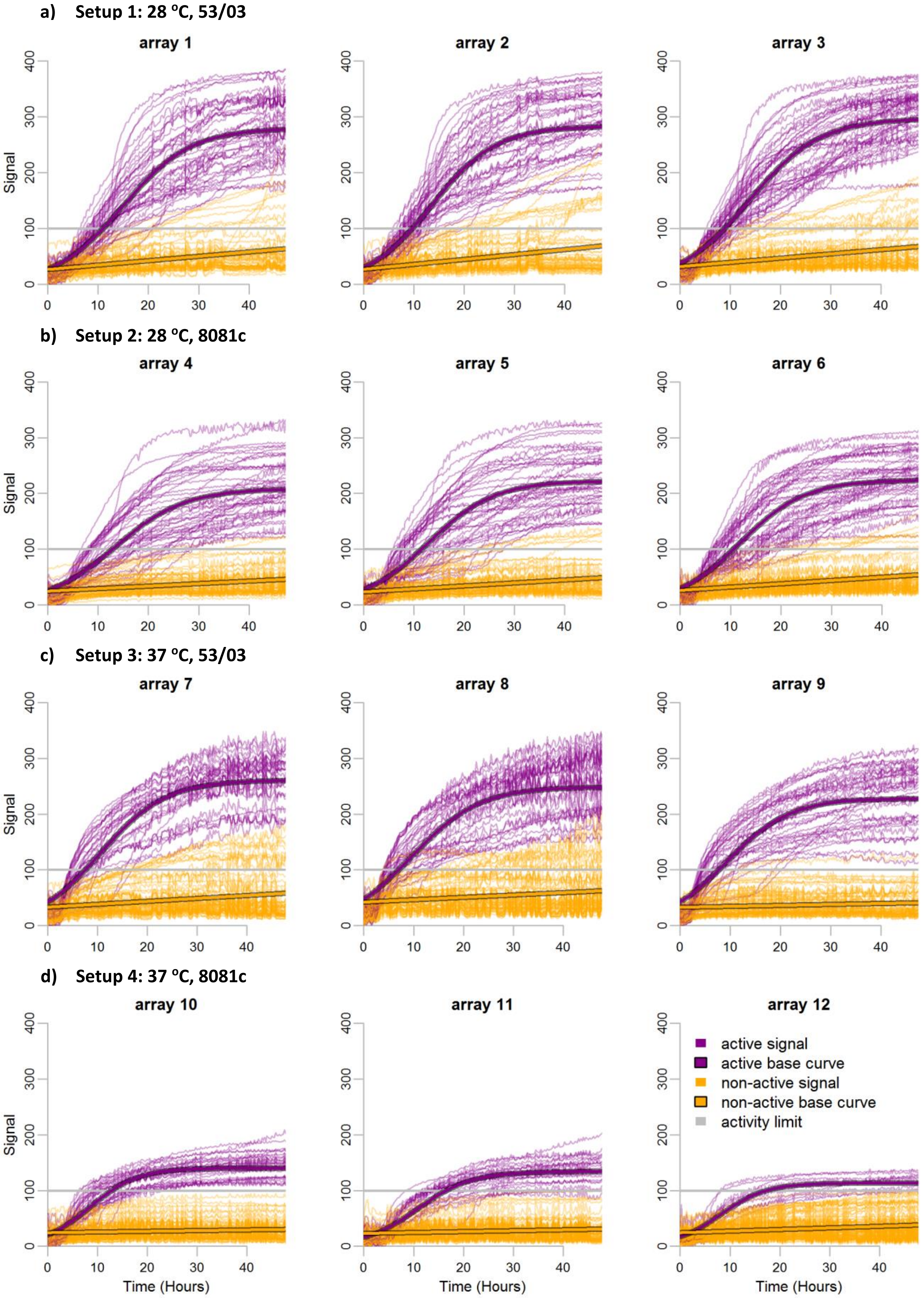
a) Setup 1: 28 °C, 53/03
array 1
array 2
array 3
b) Setup 2: 28 °C, 8081c
array 4
array 5
array 6
c) Setup 3: 37 °C, 53/03
array 7
array 8
array 9
d) Setup 4: 37 °C, 8081c
array 10
array 11
array 12
Signal
Time (Hours)
active signal
active base curve
non-active signal
non-active base curve
activity limit

Figure S3. Grouped PM profiles array-wise. Lines represent metabolic profiles of two *Yersinia enterocolitica* strains (53/03, 8081c) measured at two temperatures (28 and 37 ^{o}C) on PM01 plates. Experiment is replicated three times for each setup. (a) Setup 1: 28 ^{o}C, 53/03. (b) Setup 2: 28 ^{o}C, 8081c. (c) Setup 3: 37 ^{o}C, 53/03. (d) Setup 4: 37 ^{o}C, 8018c. Each line in a single panel represents metabolic signals detected in one of the 96 PM01 wells. Time in hours and the strength of the signals are represented on the x- and y-axes, respectively. Grouping separates active profiles from the non-active ones by using the EM algorithm. Active group is shown in purple and non-active in orange colour. Base curves illustrating the average patterns of the groups are highlighted. A threshold of 100 (shown as the grey horizontal line) is applied.
