# Supplementary material for: Novel R Pipeline for Analyzing Biolog Phenotypic Microarray Data
Source: PLoS One. 2015 Mar 18;10(3):e0118392. doi: 10.1371/journal.pone.0118392 (PMC4365023; doi:10.1371/journal.pone.0118392)
Supplement: S4 Fig — (PDF) [file pone.0118392.s004.pdf]

a) Setup 1: 28 °C, 53/03

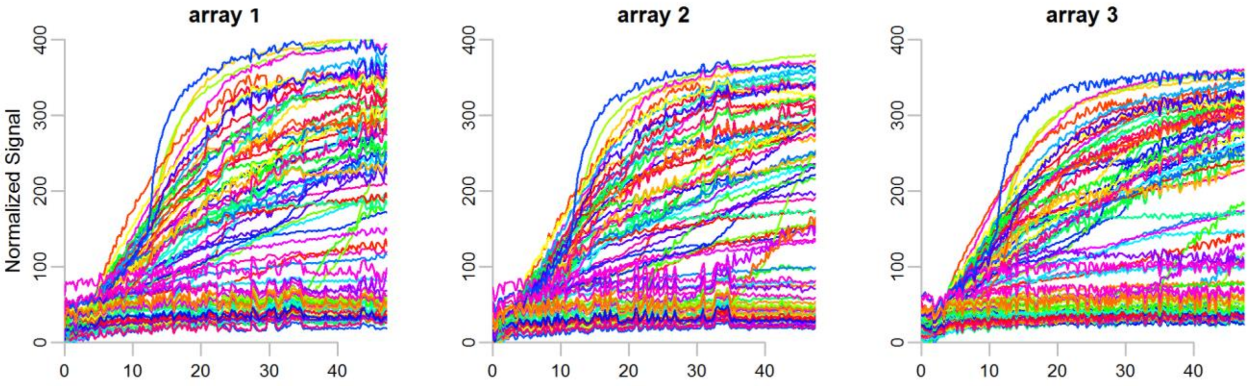

b) Setup 2: 28 °C, 8081c

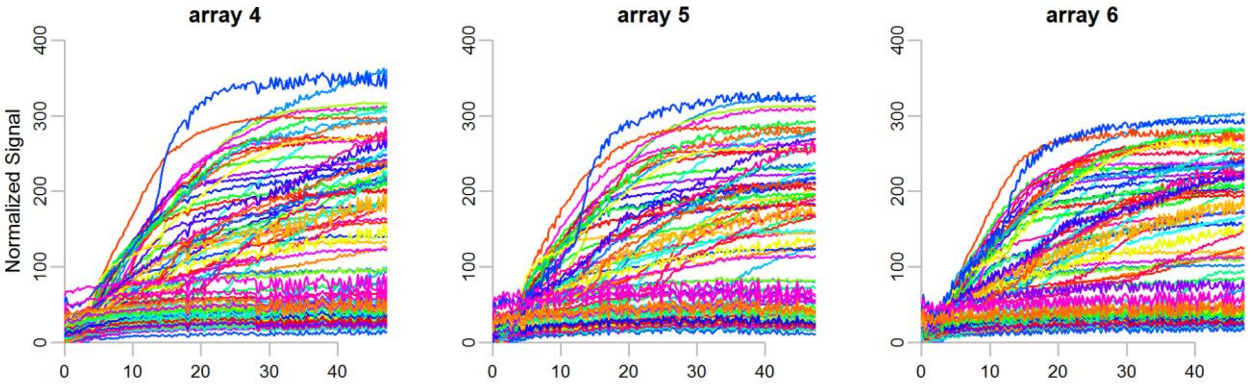

c) Setup 3: 37 °C, 53/03

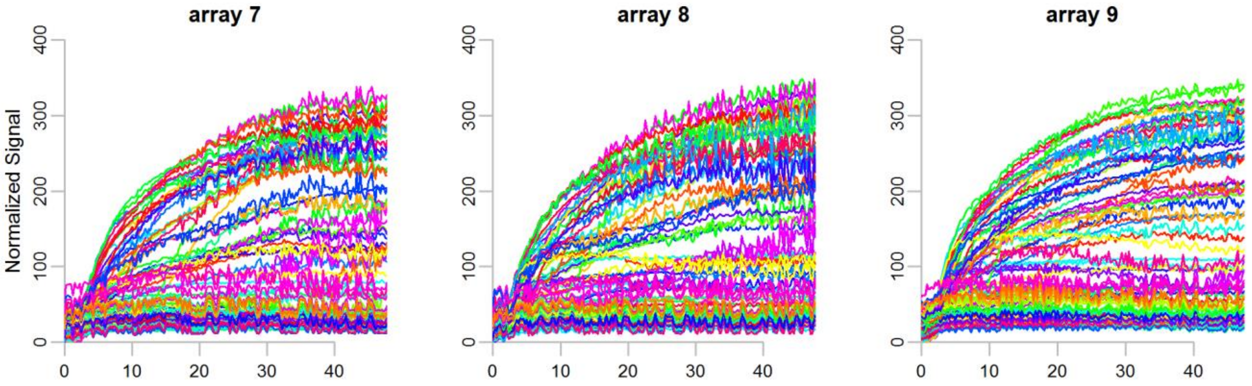

d) Setup 4: 37 °C, 8081c

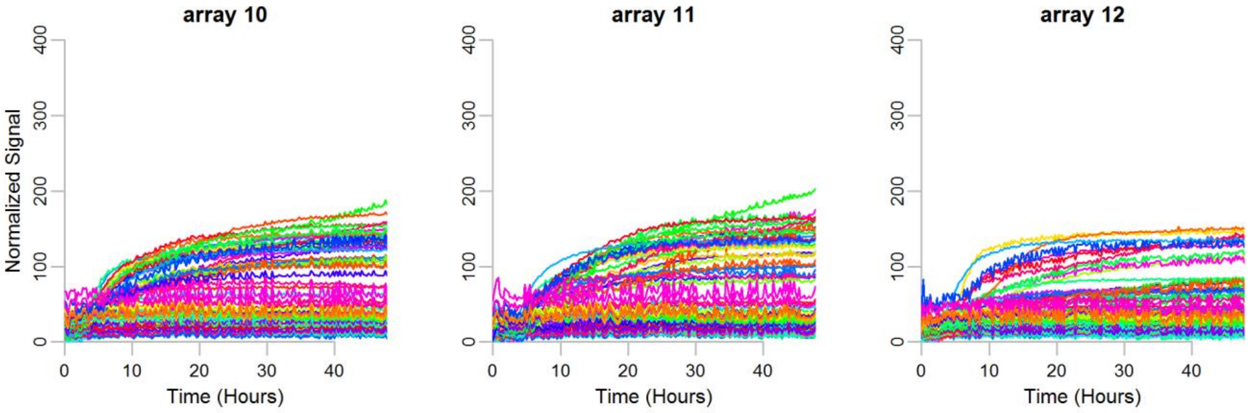

Figure S4. Normalized PM profiles array-wise. Lines represent normalized metabolic profiles of two *Yersinia enterocolitica* strains (53/03, 8081c) measured at two temperatures (28 and 37 ^oC) on PM01 plates. Experiment is replicated three times for each setup. (a) Setup 1: 28 ^oC, 53/03. (b) Setup 2: 28 ^oC, 8081c. (c) Setup 3: 37 ^oC, 53/03. (d) Setup 4: 37 ^oC, 8018c. Each line in a single panel represents one of the 96 normalized PM01 metabolic profiles. Time in hours and the normalized metabolic signals are represented on the x- and y-axes, respectively.
