# Supplementary material for: Novel R Pipeline for Analyzing Biolog Phenotypic Microarray Data
Source: PLoS One. 2015 Mar 18;10(3):e0118392. doi: 10.1371/journal.pone.0118392 (PMC4365023; doi:10.1371/journal.pone.0118392)
Supplement: S6 Fig — (PDF) [file pone.0118392.s006.pdf]

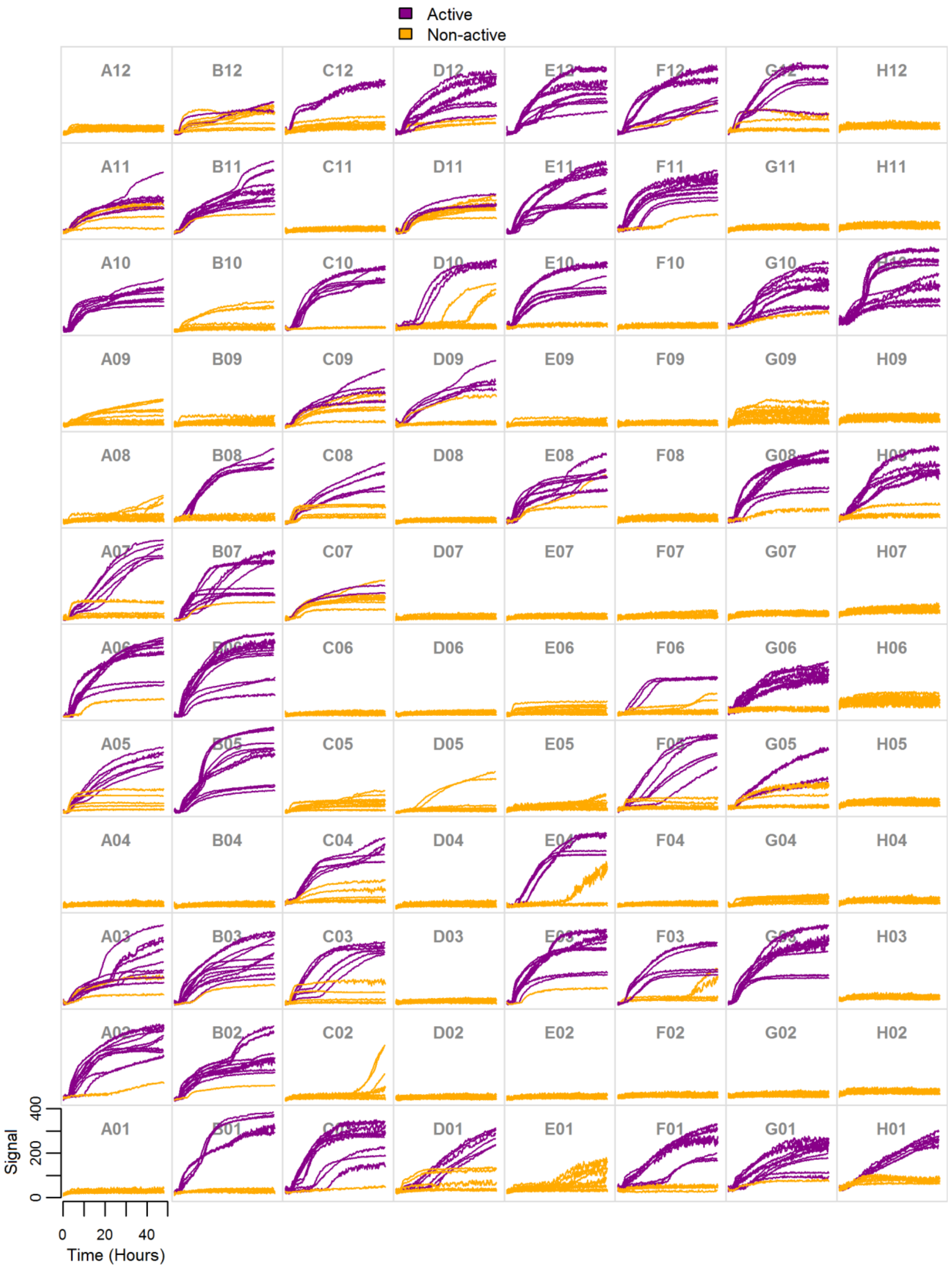

Active
Non-active
A12
B12
C12
D12
E12
F12
G12
H12
A11
B11
C11
D11
E11
F11
G11
H11
A10
B10
C10
D10
E10
F10
G10
H10
A09
B09
C09
D09
E09
F09
G09
H09
A08
B08
C08
D08
E08
F08
G08
H08
A07
B07
C07
D07
E07
F07
G07
H07
A06
B06
C06
D06
E06
F06
G06
H06
A05
B05
C05
D05
E05
F05
G05
H05
A04
B04
C04
D04
E04
F04
G04
H04
A03
B03
C03
D03
E03
F03
G03
H03
A02
B02
C02
D02
E02
F02
G02
H02
A01
B01
C01
D01
E01
F01
G01
H01
Signal
0
200
400
0
20
40
Time (Hours)

Figure S6. Grouped PM profiles substrate-wise. Lines represent metabolic profiles of two *Yersinia enterocolitica* strains (53/03, 8081c) measured at two temperatures (28 and 37 ^{o}C) on 12 PM01 plates. Each panel represents one of the 96 substrates on a PM01 plate. Time in hours and the strength of the signal are represented on the x- and y-axes, respectively. Grouping separates active profiles from the non-active ones by using the EM algorithm. Active group is shown in purple and non-active in orange colour. For the activity a threshold of 100 is applied.
