# Supplementary material for: Novel R Pipeline for Analyzing Biolog Phenotypic Microarray Data
Source: PLoS One. 2015 Mar 18;10(3):e0118392. doi: 10.1371/journal.pone.0118392 (PMC4365023; doi:10.1371/journal.pone.0118392)
Supplement: S8 Fig — (PDF) [file pone.0118392.s008.pdf]

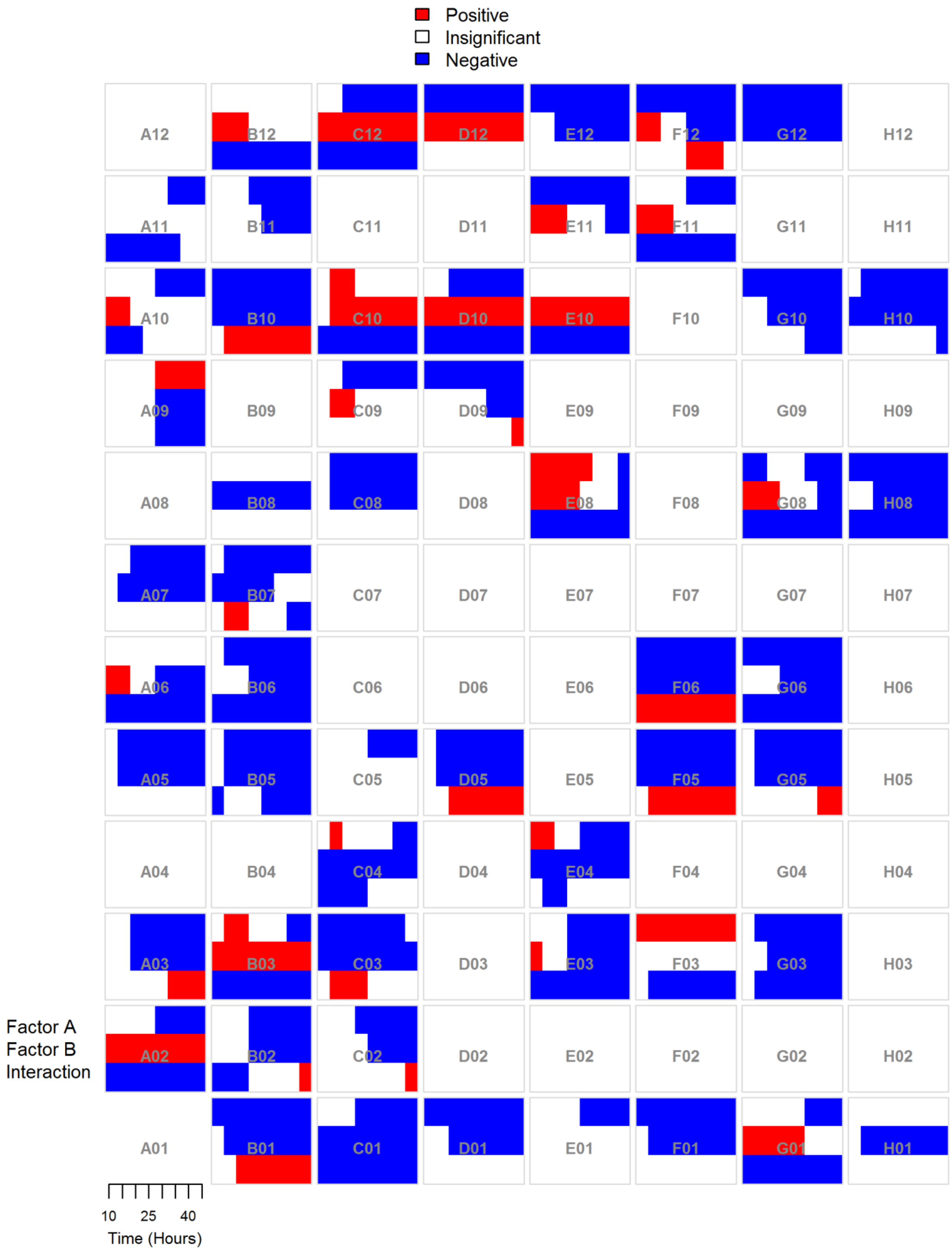

Positive
Insignificant
Negative
A12 B12 C12 D12 E12 F12 G12 H12
A11 B11 C11 D11 E11 F11 G11 H11
A10 B10 C10 D10 E10 F10 G10 H10
A09 B09 C09 D09 E09 F09 G09 H09
A08 B08 C08 D08 E08 F08 G08 H08
A07 B07 C07 D07 E07 F07 G07 H07
A06 B06 C06 D06 E06 F06 G06 H06
A05 B05 C05 D05 E05 F05 G05 H05
A04 B04 C04 D04 E04 F04 G04 H04
A03 B03 C03 D03 E03 F03 G03 H03
Factor A
Factor B
Interaction
A02 B02 C02 D02 E02 F02 G02 H02
A01 B01 C01 D01 E01 F01 G01 H01
10 25 40
Time (Hours)

Figure S8. Effect identification. Panels represent results for the effect identification using the PM measurements of two *Yersinia enterocolitica* strains (53/03, 8081c) measured at two temperatures (28 and 37 ^{o}C) on 12 PM01 plates. Each panel represents one well on a PM01 plate. Y-axis is divided into three panels, each representing one of the three effects of interest: Factor A (strain effect), Factor B (temperature effect) and their interaction. For each effect indicated is whether the effect is positive, negative or not significant. Time in hours is represented on the x-axis.
